# Impact of mental fatigue on tennis players’ attention and groundstroke performance

**DOI:** 10.3389/fpsyg.2025.1544785

**Published:** 2025-05-02

**Authors:** Cenk Öztürker, Asuman Şahan, Kemal Alparslan Erman

**Affiliations:** ^1^Institute of Medical Science, Akdeniz University, Antalya, Türkiye; ^2^Faculty of Sport Sciences, Akdeniz University, Antalya, Türkiye

**Keywords:** mental fatigue, attention, Stroop task, tennis, targeting

## Abstract

**Objectives:**

This study aimed to determine the effects of mental fatigue on attention and groundstroke targeting performance in tennis players.

**Methods:**

A total of 66 young male tennis players (age: 24.32 ± 2.46 years) participated in this randomized placebo-controlled crossover trial. Interventions included mental fatigue (MF), non-fatiguing effect (Placebo), and no mental fatigue (Control). The Stroop Attention Test (SAT) and Tennis Groundstroke Targeting Test (TGTT) depth and accuracy sections were administered before and after all interventions. Changes over time in normally distributed homogeneous data were determined using repeated-measures ANOVA (3×2), with Bonferroni correction applied for *p*-values for normally distributed variables and the Friedman test for non-normally distributed variables. Paired samples t-test and Wilcoxon test were used for pairwise comparisons of normally and non-normally distributed data, respectively.

**Results:**

Post-test pairwise comparisons showed that the MF intervention significantly increased SAT completion times and SAT error scores (*p* < 0.001). Post-intervention TGTT scores showed significant differences between MF, Placebo, and Control (*p* < 0.001), with *post hoc* analysis indicating that TGTT scores were significantly lower in the MF intervention compared to Control and Placebo (*p* < 0.001).

**Conclusion:**

This study demonstrates that acute mental fatigue decreases attention and tennis groundstroke targeting performance in male tennis players.

## 1 Introduction

Mental fatigue is a psychobiological acute state that results from extended cognitive effort, characterized by increased sensations of fatigue and low of energy (Talukdar et al., 2019; Habay et al., 2021; Fuentes-García et al., 2021).

Mental fatigue is particularly common in open-skill sports that require players to react to an unpredictable and dynamically changing environment with high cognitive demand (Sun et al., 2022).

Previous studies have determined how mental fatigue impairs technical performance (e.g., passing, dribbling, and shooting) (Moreira et al., 2018; Le Mansec et al., 2018) and physical performance (e.g., endurance in swimming, football, cycling, running, yo-yo, and volleyball) (Penna et al., 2018; Filipas et al., 2021; Fortes et al., 2021; Habay et al., 2021). Studies have also systematically synthesized the negative effects of mental fatigue on cognitive function (e.g., decision-making, attention, and perception) (Lew and Qu, 2014; Mozuraityte et al., 2023; Jaydari Fard and Lavender, 2019; Russell et al., 2019; Lopes et al., 2023) and cognitive task performance (accuracy and/or reaction time) (Verschueren et al., 2020; Habay et al., 2021; Skala and Zemková, 2022).

Tennis is an interval sport characterized by short-duration, high-intensity, repetitive movements that require anaerobic skills, such as speed, agility, and power, combined with high aerobic capacity. It is also a sport that demands cognitive skills (Tsetseli et al., 2016). Improving tennis quality and player performance can be associated with aligning technical and tactical skills with cognitive abilities (Šlosar et al., 2021).

In tennis, performance is influenced by the level of attention, and one of the most critical factors for success in tennis is the ability to direct attention to appropriate stimuli and maintain focus (Tapan et al., 2023). Wulf (2007) and Keller et al. (2021) suggested that, in tennis skill acquisition and performance, accuracy and quality depend largely on where athletes focus their attention, and focusing on the ball’s trajectory is a constant task. Higher attentional intensity allows tennis players to perceive the opponent’s movements on the court and the ball’s flight as relatively slow (Pačesová et al., 2018).

Tennis requires quick decision-making and problem-solving skills (García-González et al., 2014), so participating in intensive competitions throughout the year can create mental fatigue in sub-elite and elite level tennis players (Ding et al., 2024). Filipas et al. (2024) stated that the level of mental fatigue was an important factor in tennis performance.

To our knowledge, there has so far been limited research into the effects of mental fatigue on tennis performance. Our study, therefore, aimed to investigate the effects of mental fatigue on both attention and technical performance in tennis, specifically focusing on groundstroke. We hypothesized that mental fatigue would impair attention and reduce target performance compared with placebo and control groups.

## 2 Materials and methods

### 2.1 Participants

A total of 66 young male tennis players (age: 24.32 ± 2.46 years) voluntarily participated in this randomized placebo-controlled crossover trial.

To determine the appropriate sample size G-Power software (version 3.1.7) was used for a repeated measures analysis of variance (ANOVA) within-between interaction factors (Faul et al., 2007). Input parameters included alpha error: 0.05, power: 0.95, number of conditions: 3, number of measurements: 2, correlation between measures: 0.5, and nonsphericity correction: 1. The minimum sample size was calculated to be 66. A total of 66 participants were included in this study.

Participants who had been playing tennis for at least 3 years and scored between 50 and 75% in the Tennis Groundstroke Targeting Test (TGTT) were included. All participants were playing tennis at the intercollegiate or regional level. Participants were instructed not to consume alcohol the night before and on the test days, avoid caffeine 3 h before the tests, maintain their usual breakfast habits, and sleep at least 7 h the night before the test. They were also required to refrain from strenuous cognitive and physical activities the night before and on the day of the test. Inclusion criteria were checked before the tests, and any participant not meeting these criteria had their test days rescheduled. Exclusion criteria included participants wishing to withdraw from the study, any injury or illness, or incomplete tests. After obtaining approval from the university ethics committee, (Approval Date:10/05/2023, Protocol No: 402), all participants were given detailed information about the study and general test procedures before the tests, and informed consent was obtained.

### 2.2 Research methods

#### 2.2.1 Experiment design

The study included three different interventions: Mental Fatigue (MF), Placebo, and Control. Participants were randomly assigned to intervention groups. All participants completed the SAT and TGTT (depth and accuracy) tests for 5 min before and after each intervention. MF was induced by a 30-min Stroop task, followed by the Visual Analog Scale (VAS) to measure perceived difficulty. The placebo intervention involved reading an online sports magazine for 15 min, as it was previously used as a non-fatiguing intervention (Smith et al., 2016). The interventions were not time-matched because excessive boredom occurs after 30 min of online magazine reading. Boredom may affect performance outcomes, so the control group read an online sports magazine for 15 min (Filipas et al., 2021).

The control group had a 15-min rest between pre-and post-tests. Before the tennis tests, participants warmed up with a 5-min low-paced run and several dynamic stretching exercises. The TGTT depth and accuracy sections were conducted on a standard-sized hard court. All participants participated in all groups’ tests with at least 3 days between each test session.

#### 2.2.2 Instruments

##### 2.2.2.1 Color Stroop Test (CST)

The Stroop test identifies selective attention dysfunction and is crucial for assessing cognitive flexibility (Zalonis et al., 2009). Stroop performance can be influenced by age and education, but not by gender. It evaluates the speed at which participants can identify color names printed in different ink colors. Participants must choose the ink color of the word rather than the word itself (Ghimire et al., 2014). The Stroop task requires intense and sustained attention along with response inhibition. Participants were asked to press one of the buttons representing 4 different colors on the keyboard. The correct response is the button corresponding to the pixel color of the word. For example, if the word red appears in a green pixel, the green button should be pressed (Filipas et al., 2021). Various versions of the Stroop test exist, but all include the same color-word interference effect. The Stroop test consists of three sections: In this study, the third part of the CST was used as an attention test. The third is the color-word interference task, where participants name the color of the ink instead of the word. The test assesses the participant’s response time and error count, with the last section emphasizing the interference effect (Zalonis et al., 2009; Arsalidou et al., 2013).

CST from the Psychology Experiment Building Language (PEBL), a free access battery, was used to assess attention parameters (Mueller and Piper, 2014). CST, used as an attention test (SAT) in this research, consists of a total of 70 stimuli presented. Depending on the response time given by the participants, the average duration is between 5 and 6 min. During the test, participants are asked to distinguish and choose words and colors as quickly and accurately as possible.

Mental fatigue was induced with a 30-min computerized Stroop color-word task. The Stroop task requires intense and sustained attention along with response inhibition. Since the response times given to the stimuli during the Stroop task differ from each other, the number of stimuli presented to the players during the task varied between 363 and 479.

##### 2.2.2.2 VAS (Visual Analog Scale)

Fatigue was assessed using the VAS which is a psychometric response scale. Participants were asked the level of fatigue they felt on a scale of 1 to 10. 1 represents no fatigue and 10 represents extreme fatigue (Johnson, 2001).

Tennis Groundstroke Targeting Test (TGTT): Groundstroke depth and groundstroke accuracy tests were used to determine tennis groundstroke targeting performance [International Tennis Federation (ITF), 2004]. The ITF International Tennis Number Manual. Available online at: http://www.itftennis.com (cited December, 2023). During the test, the ball was fed by throwing it from the ball machine. The time between each hit was defined as 2 s, the amount of spin was defined as 4 units in the forward direction, and the speed of the ball was defined as 2 units.

###### 2.2.2.2.1 Test of groundstroke depth

**Figure 1:**
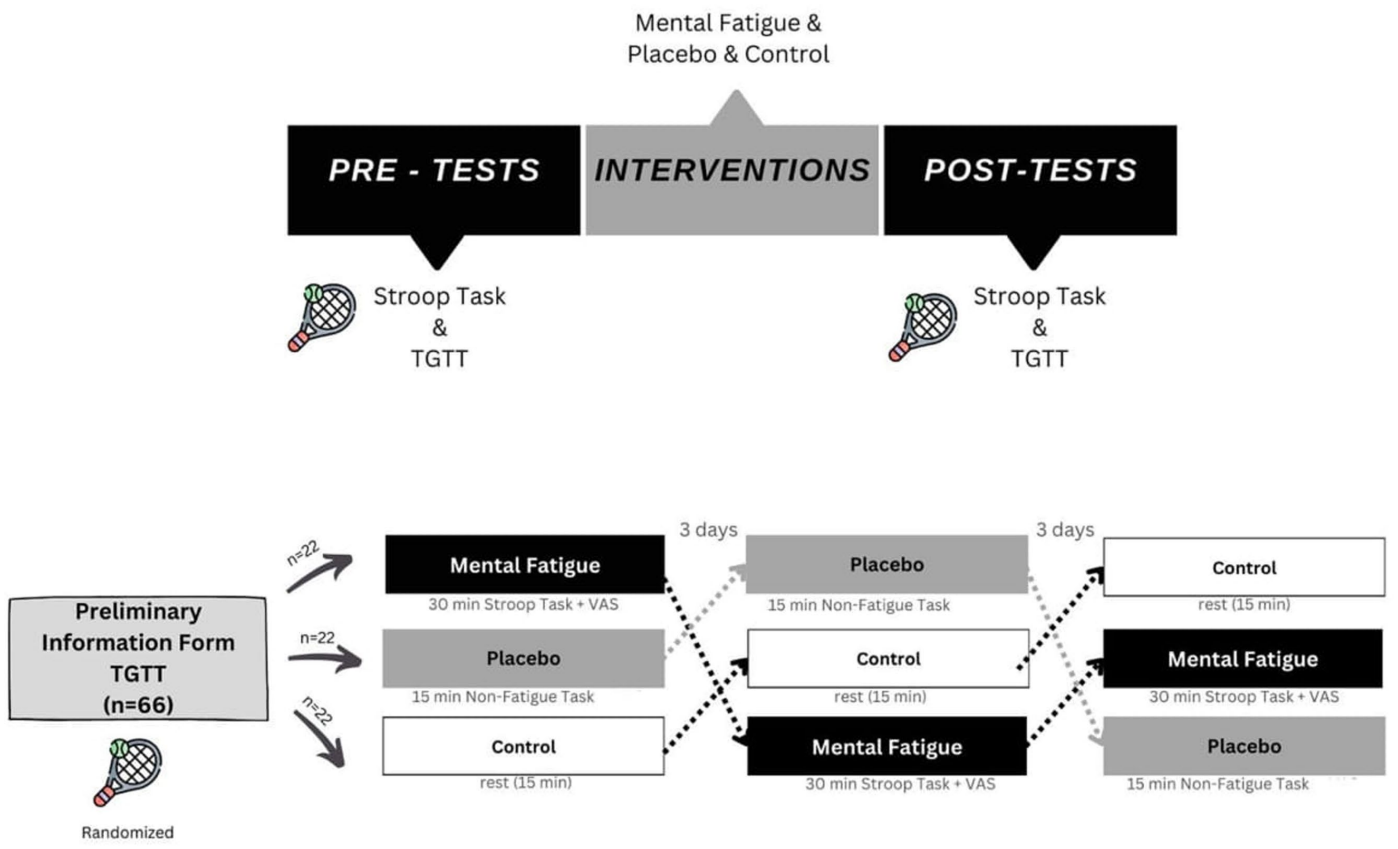
Flowchart experimental procedure.

As seen in Figure 1, the player makes 5 forehand and 5 backhand hits alternately on the balls thrown to 90×90 cm drawn targets from the ground ball throwing machine shown as P on the tennis court. The participant receives 0 points if the ball falls out or gets stuck in the net. In case the ball falls inside.

1–1, 2, 3 or 4 points depending on the region where the team falls first.

2-According to the area where it falls, if it falls within the tennis court, it will score 0 points; if it lands in the area written as 1 point extra in the power field, it will receive 1 extra point; if it lands in the area written with double points, the score it received from the area where the ball first fell will be multiplied by 2.

3–1 extra point is given for each ball that falls inside. The participant can collect a maximum of 90 points from this section.

###### 2.2.2.2.2 Test of groundstroke accuracy

**Figure 2:**
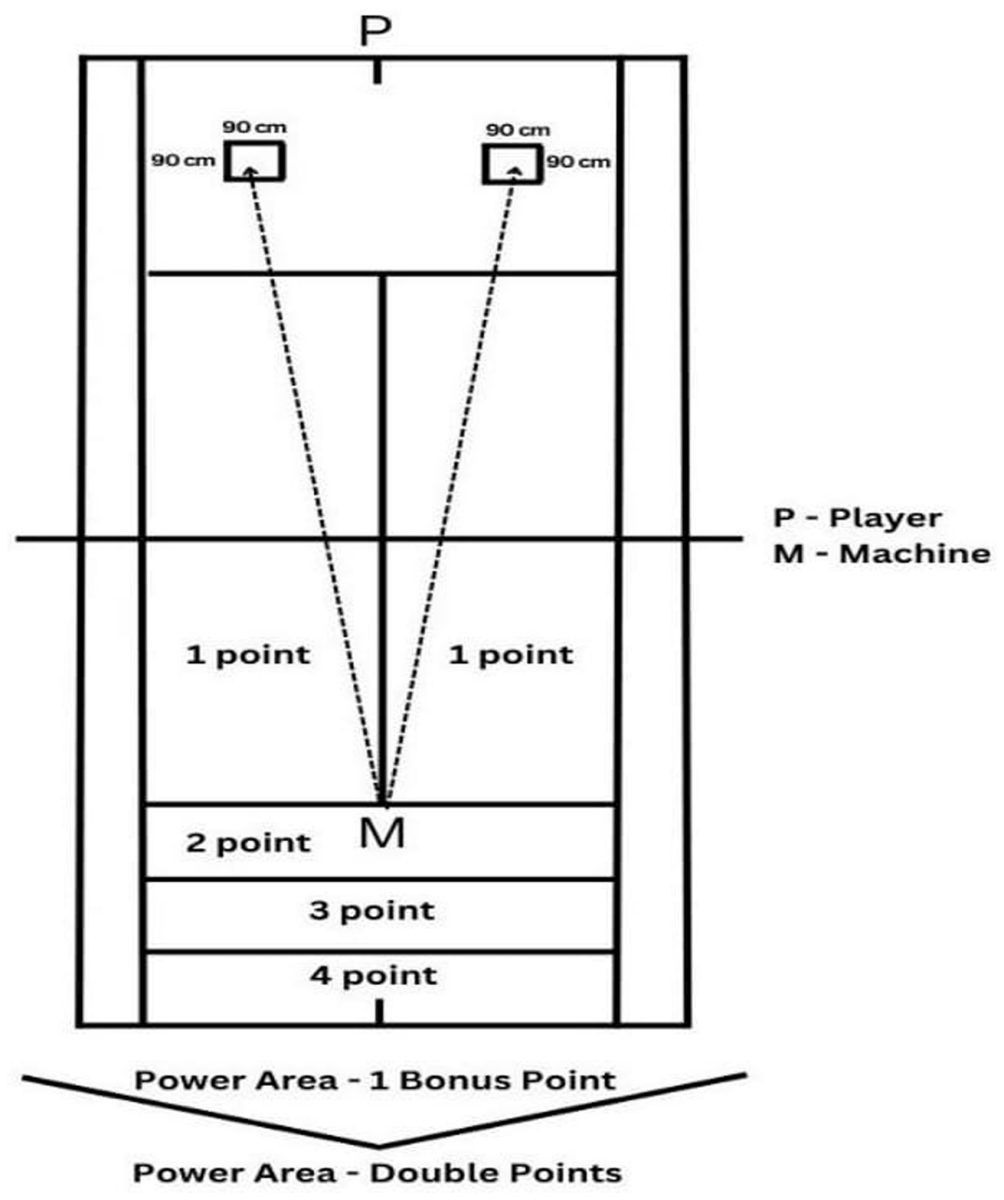
Test of groundstroke depth.

As seen in Figure 2, the player throws 6 balls alternately, one with the front hand and one with the back of the hand, to the 90×90 cm drawn targets from the ground ball throwing machine indicated as P on the tennis court. The player throws these balls parallel, then 6 more balls are thrown to the same targets alternately with the front of the hand and the back of the hand, and the player throws these balls diagonally.

**Figure 3:**
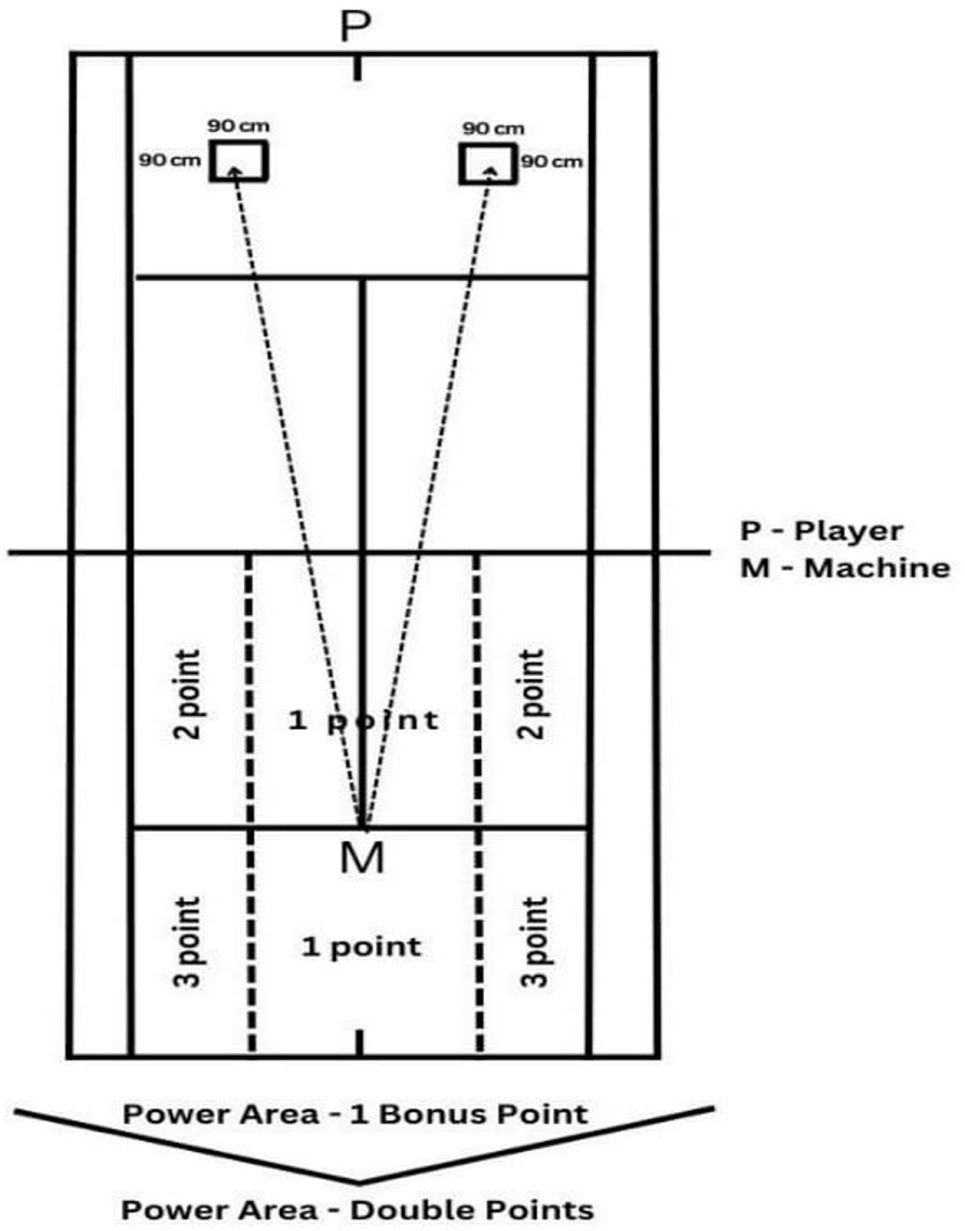
Test of groundstroke accuracy.

The participant receives 0 points if the ball gets stuck in the net or falls outside the playing field. In case of falling inside, the participant gets 1, 2 or 3 points depending on the area where he fell first, 0 points if he falls inside the playing field depending on the area, he fell 2nd, and 1 extra point if he falls in the area written as force field - 1 extra point. If the ball lands in the force area - Double points written area, the score from the first area will be multiplied by 2. 1 extra point is awarded for each ball that falls in. The participant can collect a maximum of 84 points from this section (Figure 3).

### 2.3 Data analysis

The normality of data was assessed using the *Shapiro–Wilk test*, with descriptive statistics (mean, standard deviation) calculated for normally distributed variables. *Repeated-measures ANOVA* was used to determine changes over time (3×2), with *Bonferroni* correction for *post hoc comparisons*. The *Friedman test* was used for non-normally distributed variables, with *Wilcoxon signed-rank tests* and *Paired Samples t Test* for pairwise comparisons. The significance level was taken as *p* < 0.05 and *p* < 0.01. The effect size was evaluated as *η*2 = 0.01 low, *η*2 = 0.06 medium, and *η*2 = 0.14 large effect level (Cohen, 1988). We performed statistical analyses using SPSS Statistics ver. 28 (IBM Corp. NY, Armonk, United States), with a significance level of 5%.

## 3 Results

### 3.1 Baseline characteristics

Mean±SD age of male subjects was 24.32 ± 2.46 years (range, 18 to 25 years). Mean±SD values for body weight, height and BMI were 74.64 ± 8.01 kg (range, 60 to 104 kg), 178.05 ± 5.82 cm (range, 165 to 190 cm) and 23.51 ± 1.86 kg/m2 (range, 19.66 to 29.42 kg/m2), respectively.

Data in the table; The mean ± standard deviation (Mean ± SD) is presented.

According to the results of Friedman and Wilcoxon analysis **p < 0.01.

When Table 1 was examined, no significant differences in SAT completion times and error scores between interventions before the tests (*p* > 0.05). Significant differences were found in SAT completion times and error scores between groups after the interventions (*p* < 0.001).

**Table 1:** SAT durations before and after interventions (min).

Intervention	Pre-test	*z*	*p*	Post-test
Mental Fatigue	18.08 **±** 11.96	−4.02	0.001**	23.05 ± 11.31
Placebo	19.23 **±** 11.94	−1.88	0.06	16.68 ± 11.36
Control	20.14 **±** 11.97	−1.53	0.13	18.05 ± 12.38
	*X*^2^ = 1.55 *p* = 0.46			*X*^2^ = 10.90 *p* = 0.001**

Post-test pairwise comparisons showed that the MF intervention significantly increased SAT completion times compared to Control (*p* = 0.001) and Placebo (*p* = 0.001) interventions (p < 0.001), with no significant difference between Placebo and Control (*p* > 0.05).

In pre-posttest comparisons between interventions, it was determined that there was a significant increase only in the MF intervention (*p* = 0.001), (*p* < 0.001).

Data in the table; The mean ± standard deviation (Mean ± SD) is presented.

According to the results of Friedman and Wilcoxon analysis *p < 0.05; **p < 0.001.

When Table 2 was examined, no difference was found in the error scores before the interventions in the attention parameter (*p* = 0.46). A significant difference was found between error scores after the interventions (*p* = 0.001).

**Table 2:** SAT error scores before and after interventions.

Intervention	Pre-test	*z*	*p*	Post-test
Mental fatigue	5.70 ± 0.24	−3.92	0.001**	5.84 ± ±0.24
Placebo	5.71 ± 0.23	−0.14	0.89	5.73 ± 0.25
Control	5.68 ± 0.22	−0.89	0.37	5.68 ± 0.23
	*X*^2^ = 1.09 *p* = 0.57			*X*^2^ = 9.84 *p* = 0.001**

Post-test pairwise comparisons of SAT error scores showed that the MF intervention significantly increased error scores compared to Placebo (*p* = 0.001) and Control (*p* = 0.01), with no significant difference between Control (*p* = 0.13) and Placebo (*p* = 0.64).

In pre-posttest comparisons between interventions, it was determined that there was a significant increase only in the MF intervention (*p* = 0.001).

Data in the table; The mean ± standard deviation (Mean ± SD) is presented. η2 partial eta squared (effect size) 0.01 < low, 0.06 < −medium,0.14 < large; According to Repeated Measurement Anova and Paired samples t test results *p < 0.05; **p < 0.001.

According to Table 3, a significant difference was found between the interventions in the pre-and post-tests (*p* < 0.01). According to the *post hoc* analysis results performed in the pre-tests, it was determined that the TGT (depth, precision) pre-test total scores were significantly higher in the MF intervention than in the Control and Placebo interventions (*p* = 0.001), (*p* < 0.001). There was no significant difference between Placebo and Control (*p* = 0.34) (*p* > 0.05). According to the results of post hoc analysis on total scores in the post-tests, it was determined that the MF intervention group was significantly lower than the Control and Placebo interventions (*p* = 0.001), (*p* < 0.001). There was no significant difference between Placebo and Control (*p* = 0.70) (*p* > 0.05).

**Table 3:** TGTT total scores before and after intervention.

Intervention	Pre-test	*t*	*p*	Post-test
Mental fatigue	98.80 ± 13.99	11.09	0.001**	83.70 ± 16.63
Placebo	93.61 ± 15.97	1.43	0.16	91.77 ± 15.79
Control	90.33 ± 15.57	−2.99	0.001**	94.39 ± 13.43
	F_(2;130)_ = 10.32 *p* = 0.001** η2 = 0.014			*F*_(2;130)_ = 16.46 *p* = 0.001** η2 = 0.20

In the pre-posttest comparisons between interventions, it was determined that there was a significant decrease (*p* = 0.001) in the MF intervention, while there was a significant increase (*p* = 0.001) in the measurements without intervention (Control) between the tests (*p* < 0.001). There was no significant difference in the placebo group (*p* = 1.43) (*p* > 0.05).

The VAS result used to determine whether mental fatigue was successfully induced after the 30-min Stroop task applied to the participants was found to be 5.27 ± 1.20.

## 4 Discussion

This randomized, placebo-controlled, crossover study examined the effects of mental fatigue on male tennis players’ attention and groundstroke targeting performance. The attention test durations and error scores increased and total tennis targeting scores decreased with the mental fatigue intervention compared to the placebo and control interventions. Mental fatigue was shown to impair attention and reduce groundstroke performance.

Attention, one of the cognitive features central to tennis, not only increases learning and performance, but also increases movement efficiency (Tsetseli et al., 2016).

Prolonged application of the Stroop task ––a neuropsychological test of attention and reaction ability (Scarpina and Tagini, 2017) –– causes mental fatigue (Cuchna et al., 2024). In our study, attention duration and error scores were found to be similar before the Stroop task but were significantly different after the task. There was no significant change for the control and placebo interventions. The total tennis targeting scores decreased significantly decreased with mental fatigue intervention.

Studies investigating the effects of mental fatigue on cognitive performance and skill performance in games such as football, basketball, and table tennis has been limited (Ding et al., 2024). To the best of our knowledge, only a few studies have investigated the effects of mental fatigue on accuracy. Studies conducted among basketball players (Moreira et al., 2018), football players (Filipas et al., 2021), and table tennis players (Yann Le Mansec et al., 2018) have shown that mental fatigue impairs targeting performance. In a study involving a small sample group of tennis players, it was determined that mental fatigue increased the percentage of failed second serves from the deuce under mental fatigue conditions, while no significant differences between mental fatigue and control groups were found in first and second-serve speed or accuracy (Filipas et al., 2024).

Fuentes-García et al. (2021) found that mental fatigue reduced serve speed. Our study, however, aimed to determine the effect of mental fatigue on attention, which is one of the cognitive characteristics critical for tennis performance, and groundstroke targeting performance, which is one of the most frequently used techniques.

Marcora et al. (2009) proposed a psychobiological model of endurance performance, suggesting that mental fatigue triggered activation of the anterior cingulate cortex (ACC) and, therefore, adenosine levels increased, and dopamine transmission decreased. They stated that mentally tired players perceived effort more intensely and therefore performance decreased. Considering the role of the ACC in attention regulation, performance decreases caused by mental fatigue may result from inadequate allocation of cognitive resources to attention (Ding et al., 2024).

In the present study, mental fatigue appeared to reduce hand-eye coordination, an important element for contacting the ball at the right time and place during groundstroke targeting. This suggests that mental fatigue affects attentional focus processes as well as motor processes.

## 5 Limitations and avenues for future

Our study used the VAS self-assessment test to assess mental fatigue, reducing the sensitivity of our results. Future research could use fatigue assessments together with VAS. Our study was also not able to test the hypotheses with advanced players and this could be another fruitful avenue for future research. Our test subjects were all men and it would be interesting to carry out the research with female players to see whether their technical performance was similarly affected by mental fatigue.

Future studies could also research the effects for different age groups, and ability levels. Comprehensive research is also needed on strategies to counteract performance decline due to mental fatigue.

## 6 Conclusion

Our study revealed that mental fatigue led to lower tennis targeting performance and impaired attention compared to placebo and control interventions, highlighting the negative effects on both attention and motor skills. Tennis players and coaches are advised to avoid intensive cognitive tasks before competitions and to seek ways to prevent mental fatigue.

## Data Availability

The raw data supporting the conclusions of this article will be made available by the authors, without undue reservation.
